# *TNFRSF13B/TACI* Alterations in Turkish Patients with Common Variable Immunodeficiency and IgA Deficiency

**Published:** 2018

**Authors:** Neslihan Edeer Karaca, Ezgi Ulusoy Severcan, Burcu Guven, Elif Azarsiz, Guzide Aksu, Necil Kutukculer

**Affiliations:** Department of Pediatric Immunology, Faculty of Medicine, Ege University, Izmir, Turkey

**Keywords:** Common variable immunodeficiency, IgA deficiency, TACI, Mutation, Respiratory tract infection

## Abstract

**Background:**

The Transmembrane Activator and Calcium modulator ligand Interactor (TACI), encoded by *TNFRSF13B/TACI* gene, is mutated in some patients with Common Variable Immunodeficiency (CVID) and IgA Deficiency (IgAD). The purpose of the study was to investigate for the first time in Turkish patients the prevalence of *TNFRSF13B* alterations in CVID, selective and partial IgAD patients.

**Methods:**

Forty two CVID, 36 selective IgAD, 34 partial IgAD and 25 healthy controls were included. All patients were examined for *TNFRSF13B* gene mutations by PCR.

**Results:**

The percentages of *TNFRSF13B* mutations in CVID, selective and partial IgAD patients were 7.1, 2.7 and 2.9%, respectively. No disease causing *TNFRSF13B* mutation in healthy controls was found. Patients with TACI mutations had recurrent respiratory tract infections. None of them experienced autoimmunity, bronchiectasis or granulomatous disease. In conclusion, *TNFRSF13B* mutations were present not only in CVID patients, but also in IgAD cases.

**Conclusion:**

Modifier genes as well as their combination with other genetic or environmental factors may play an important role in the development of the immunodeficiency phenotype.

## Introduction

Defects in antibody production predispose antibodies to various types of infections that are the hallmark of Primary Antibody Deficiencies (PADs). IgA Deficiency (IgAD) and Common Variable Immunodeficiency (CVID) are the most prevalent PADs in humans, with an estimated incidence of 1:600 and 1:25000, respectively 1,2. Although most cases of IgAD and CVID are sporadic, there is a family history in about 20–25% of cases. A common genetic basis for IgAD and CVID is its incidence in members of the same family and similarity of the underlying B cell differentiation defects. Additionally, some cases initially present with IgAD which later evolves into CVID.

The Transmembrane Activator and Calcium modulator ligand Interactor (TACI), encoded by *TNFRSF-13B/TACI* gene, is a TNF receptor superfamily member expressed on B cells. TACI activates IgG and IgA class switch recombination in B cells and mediates immunoglobulin (Ig) production. It is also important in regulation of B-cell homeostasis, and the antibody response to T-independent antigens. TACI is mutated in up to 10% of CVID patients. Mutations C104R, A181E, C76R, R202H were proven to be disease-causing 2. Most index patients were heterozygous, and their pedigree was consistent with autosomal dominant inheritance. In addition to these mutations, there are lots of Single Nucleotide Polymorphisms (SNPs) such as V220A, T27T, S277S and P251L, which can be observed both in patients and healthy controls. There are few studies about TACI defects in IgA deficiency cases. In these studies, the ratio of mutations differs from 0 to 16% 3,4.

The aim of this study was to clarify, for the first time in Turkish patients, the prevalence of *TNFRSF-13B* alterations in CVID, selective and partial IgAD patients.

## Materials and Methods

Forty-two CVID, 36 selective IgAD, 34 partial IgAD and 25 healthy controls were included. All of the patients fulfilled the ESID/PAGID diagnostic criteria 5. Selective IgAD is defined as an IgA level that is less than 7 *mg/dl*. Partial IgAD is defined as IgA level which is lower than mean-2SD of age-related normal levels. Clinical information and some laboratory data were obtained for all patients from their medical records. Flow cytometry analysis was performed on a FACSCalibur instrument (BD Biosciences) by using Cell Quest (BD) software. CVID patients were classified according to the EuroClass classification. The coding sequence of *TNFRSF13B* gene was amplified by PCR, and each PCR product was then sequenced directionally using the Sanger sequencing methodology. The SPSS-16 was used for statistical analysis.

## Results

Demographic characteristics of the study group and Ig levels at diagnosis are shown in table 1. The consanguinity rate was significantly higher in the CVID group (p=0.003). Recurrent Respiratory Tract Infections (RTIs) were the main clinical manifestations in all groups. The frequency of complications observed in CVID patients is listed in table 1. None of the complications such as bronchiectasis, autoimmunity, splenomegaly, granulomatous disease or malignancy were observed in IgAD patients.

**Table 1.** Demographic and clinical features and *TNFRSF13B/TACI* alterations in the study group

	**CVID (n=42)**	**Selective IgAD (n=36)**	**Partial IgAD (n=34)**	**IgAD (n=70)**	**Healthy controls (n=25)**
**Gender**	10 girls, 32 boys	14 girls, 22 boys	15 girls, 19 boys	29 girls, 41 boys	12 girls, 13 boys
**Consanguinity**	34.4%	8.3%	5.9%	7.2%	16.1%
**Family history**	19.4%	(-)	20.6%	10.4%	(-)
**Age (months)**	101.3±53.4	65.2±44.7	69.1±42.1	67.1±43.2	72.4±51.8
**IgG (*mg/dl*)**	432.3±142.2	1242.5±509.2	1005.5±325.9	1132.2±479.1	986.2±157.2
**IgM (*mg/dl*)**	65.4±63.5	110.2±79.6	102.3±16.1	106.8±62.6	105.8±40.8
**IgA (*mg/dl*)**	25.7±23.2	<6	23.7±16.1	17.1±15.9	91.9±37.4
**Autoantibody positivity**	18.5%	21.7%	16.7%	22.1%	2.4%
**Clinical data**					
Recurrent upper respiratory tract infections	35.5%	94%	82.4%	83.1%	(-)
Recurrent bronchopneumonia/pneumonia	83.9%	5.6%	5.9%	5.7%	(-)
Bronchiectasis	50%	(-)	(-)	(-)	(-)
Chronic sinusitis	66.7%	(-)	(-)	(-)	(-)
Splenomegaly	38.9%	(-)	(-)	(-)	(-)
Autoimmune clinical manifestation	11.8%	(-)	(-)	(-)	(-)
Granulomatous infiltration (lung, liver)	7.1%	(-)	(-)	(-)	(-)
Malignancy	5.6%	(-)	(-)	(-)	(-)
**Frequency of mutations in TNFRSF13B/TACI**	7.1% (n=3)	2.7% (n=1)	2.9% (n=1)	2.8% (n=2)	(-)
**Type of *TNFRSF13B/TACI* mutations**	C104R heterozygous (n=2) C204–205insA (n=1)	R202H heterozygous (n=1)	C104R heterozygous (n=1)	R202H heterozygous (n=1) C104R heterozygous (n=1)	
**SNPs in *TNFRSF13B/TACI***	40%	25%	8.8%	17%	20%
**Type of SNPs in *TNFRSF13B/TACI***	P251Lheterozygous/T27Thomozygous (n=3) V220A heterozygous (n=1) S277S heterozygous (n=2) T27T heterozygous (n=5) T27T homozygous (n=5) S277S homozygous (n=2) T27T heterozygous/S277S heterozygous (n=4)	T27T heterozygous (n=5) T27T heterozygous/S277S heterozygous (n=4)	V220A heterozygous (n=1) S277S heterozygous (n=2)	T27T heterozygous (n=5) T27T heterozygous/S277S heterozygous (n=4) V220A heterozygous (n=1) S277S heterozygous (n=2)	P251L heterozygous (n=2) P251L homozygous (n=1) S277S homozygous (n=2)

The percentages of *TNFRSF13B* mutations and SNPs in CVID patients were 7.1% and 40%, respectively. Two patients with 310T→C transition in exon 3 of the *TNFRSF13B* gene that resulted in a cystein 104 to arginine (c.310T>C, p.C104R) substitution in the extracellular domain of TACI and one patient with c.204_205insA, p.L69TfsX12 in exon 3 of the *TNFRSF13B* gene mutation were identified. The SNPs are listed in table 1.

**Figure 1.**
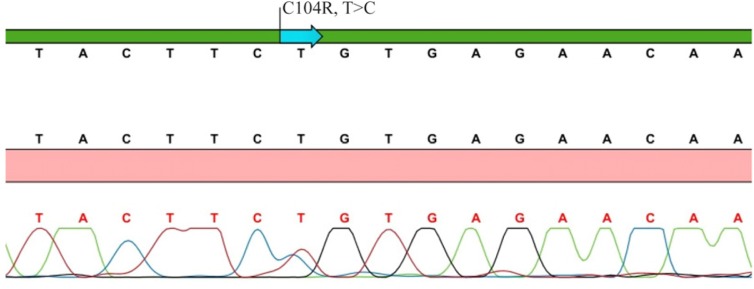
Sanger sequence chromatogram view of *TNFRSF13B/TACI* gene C104R (T>C) mutation of patient 1.

The first patient (P.1) carrying *TNFRSF13B*C104R mutation presented with RTI and lymphoproliferation at the age of 8 years. Sanger sequence chromatogram view of *TNFRSF13B* gene C104R (T>C) mutation of P. 1 is shown in figure 1. He had low Ig levels (IgG 503 *mg/dl*, IgM 18, IgA 26 *mg/dl*) with inadequate specific polysaccharide antibody responses. According to the Euroclass classification, he was in the smB^−^, CD21^norm^ group. He is doing well under regular Ig replacement therapy without any severe complications. The other patient with *TNFRSF13B* C104R mutation had frequent sinopulmonary infections since he was 12 years of age. He required hospitalization twice for pneumonia. He had low IgG (670 *mg/dl*) and IgM (16 *mg/dl*) with normal IgA levels. He did not have any serious infections or complications after diagnosis.

A twenty-four year old patient with *TNFRSF13B-*c.204_205insA mutation had recurrent infections beginning at the age of 8 years and developed acute myeloid leukemia at the age of 14 and was recovered with chemotherapy. He was in the Euroclass smB^+^, CD21 ^low^ group. He is also well with subcutaneous Ig replacement since then.

The frequency of TACI mutations and SNPs in the selective IgAD patients was 2.7% and 25%, respectively (Table 1). A four year old boy was admitted with complaints of RTI and acute otitis media for 2 years. He was found to have a heterozygous 605G-A transition in exon 4 of the *TNFRSF13B* gene that caused an arginine 202 to histidine (c.605G>A, p.R202H) substitution in the intracellular domain of the TACI protein.

One *TNFRSF13* mutation and 3 SNPs were identified in the partial IgAD group. The patient with *TNFRSF13B* C104R mutation was an 11 year old boy with the history of RTI and otitis media for one year.

All patients who carried *TNFRSF13B* mutations were born to non-consaguineous healthy parents. None of the patients with *TACI* defects experienced autoimmunity, bronchiectasis or granulomatous disease. A total of 25 healthy controls were analyzed for sequence variants in *TNFRSF13B*. No disease causing *TNFRSF13B* mutation was found. The frequency of TACI SNPs was 20%.

## Discussion

CVID is a heterogeneous primary immunodeficiency and the search for causative or susceptibility gene(s) has been progressing for the last two decades. Rare autosomal mutations in single genes, named as TACI, ICOS, BAFF-R, CD19, CD20, CD81 and MSH5, have recently been reported in CVID 1,5. Despite being so common, the exact molecular cause for IgAD is still unknown; it has been associated with HLA, particularly with the HLA A*0101: Cw*0701: B*0801: DRB1* 0301: DQA1*0501: DQB1*0201 and mutations in genes *TACI, BAFF-R, APRIL, CTLA-4, ICOS* and *RAG1*^1^.

In the literature, the frequency of disease causing *TNFRSF13B* mutations is reported to be 4.8–21% in CVID and 2.7–16% in IgAD 2–4,6. Two mutations, C104R and A181E, have a more profound effect and are more prevalent in CVID patients compared to controls. In our study, the frequency of *TNFRSF13B* alterations was 7.1% in CVID, 2.7% in selective IgAD and 2.9% in partial IgAD. C104R heterozygous mutation was detected in 3 patients. Single cases were found to carry c.204_205insA and R202H mutations. None of the patients had A181E mutation. Forty percent of CVID patients and 20% of controls carried *TNFRSF13B* SNPs, namely P251L, T27T, V220A and S277S in heterozygous, homozygous or compound heterozygous states. According to the previously reported information, these variants have been regarded as benign polymorphic variants.

TACI is essential for the establishment of central B-cell tolerance, given that all subjects carrying C104R and A181E missense *TNFRSF13B* mutations have an inability to remove developing autoreactive B cells in bone marrow 2,4. There is a general agreement that monoallelic mutations are associated with autoimmunity and lymphoproliferation phenotype in CVID. Two CVID patients and 1 IgAD patient carried C104R mutation and no association was observed between *TNFRSF13B* mutations and autoimmune manifestations in our Turkish patients. One of them has generalized lymphadenopathy which regressed after the initiation of regular Ig therapy.

Martinez-Gallo *et al* investigated the expression and function of TACI mutations in healthy heterozygous, normogammaglobulinemic relatives of subjects with CVID, who had *TNFRSF13B* mutations, and the authors showed that healthy relatives had also *in vitro* B cell defects 6. They concluded that impaired TACI signaling could not be the single defect of CVID pathogenesis.

## Conclusion

The observation that *TNFRSF13B* mutations are present not only in CVID patients, but also in IgAD cases suggests that modifier genes as well as their combination with other genetic or environmental factors may play an important role in the development of the immunodeficiency phenotype. CVID is not commonly found in more than one member of a family, for about 10% of other first-degree relatives may be either hypogammaglobulinemic or may have selective IgA deficiency. So, IgA deficiency and CVID have been known to coexist in families.

Clinicians should be aware of the fact that the interpretation of genetic testing for TACI mutations is difficult for risk assessment and the potential impact on clinical management is still limited. Well-designed investigations including a greater number of patients are necessary to make clear interpretations of the disease-causing effects of genetic alterations, their clinical manifestations and their contribution to the selection of treatment strategies. All in all, this study is the first one for identifying the the prevalance of *TNFRSF13B* alterations in Turkish patients with CVID and IgAD and may raise awareness about the complex complications in mild IgAD.
